# Yogurt Products Fortified with Microwave-Extracted Peach Polyphenols

**DOI:** 10.3390/gels9040266

**Published:** 2023-03-23

**Authors:** Athina Theocharidou, Evdoxios Psomas, Antonios Koliouskas, Christos Ritzoulis

**Affiliations:** 1 Department of Food Science and Technology, International Hellenic University, Alexander Campus, 57400 Thessaloniki, Greece; 2 Department of Hygiene and Technology of Food of Animal Origin, Veterinary Research Institute, Hellenic Agricultural Organization-Demeter, Campus of Thermi, 57001 Thessaloniki, Greece; 3 Koukakis Farm S.A., Kato Apostoli, 61100 Kilkis, Greece

**Keywords:** yogurt, peach, polyphenols, pectin, texture analysis, experimental design

## Abstract

Pectin and polyphenols have been obtained from choice peach flesh using microwave extraction, with the resulting extracts used in functionalizing strained yogurt gels. A Box-Behnken design was utilized in order to co-optimize the extraction process. Soluble solid content, total phenolic content, and particle size distributions were measured in the extracts. Extraction at pH 1 yielded the highest phenolic content, while increases in the liquid-to-solid ratio resulted in a decrease in soluble solids and an increase in particle diameter. Selected extracts were then incorporated into strained yogurt, and the resulting gel products were assessed for color and texture over a two-week period. All samples were darker and had more red tones than the control set yogurt, while exhibiting less yellow tones. The cohesiveness of all samples remained stable over the gels’ aging of two weeks (break-up times always remaining within 6 s and 9 s), which is close to the expected shelf-life of such products. The work required for the deformation of most samples increases with time, indicating that the products became firmer due to the macromolecular rearrangements in the gel matrix. The extracts obtained with the highest microwave power (700 W) give less firm samples. This was due to the microwave-induced loss of conformation and self-assembly of the extracted pectins. The hardness of all samples increased over time, gaining from 20 to 50% of the initial hardness due to the rearrangement of the pectin and yogurt proteins over time. The products with pectin extracted at 700 W were again exceptions, losing hardness or remaining stable after some time. Overall, this work combines the sourcing of polyphenols and pectin from choice fruit; it uses MAE for isolating the materials of interest; it mechanically examines the resulting gels; and it performs all the above under a specifically-set experimental design aiming towards optimizing the overall process.

## 1. Introduction

Peach (*Prunus persica*) is among the most varied fruits species assuming different sizes, flesh types, skin types, and different shapes. It is cultivated mainly in China, followed by Italy and Spain [1]. Peaches contain a mixture of polyphenols, such as phenolic acids, anthocyanins, flavonols, and flavanols [2], and they are a good source of vitamins A, B, and C [1]. The chemical composition of peaches, as with other fruits, depends on factors, such as the maturity stage, the cultivar, and storage conditions. [1]. They consist of approximately 90% water and are rich in both micro and macro- nutrients with low amount of lipids, sugars, and organic acids [1].

Peach is one of the most popular fruits worldwide, and it is used also in the production of other products, such as tea, jellies, and sweets. In the food industry, it is used to create peach puree, syrup, juice, jams, and yogurt, among other products [3,4]. However, excessive usage and consumption of peaches can result in a large amount of lower grade fruits and by-products which are often used as animal feed, burned, or disposed in landfills. Due to the high nutritional value of peaches, which contain substances such as vitamins, pectin, and phenolic compounds, new strategies have been developed for the better utilization of peach by-products. These strategies aim to reduce environmental pollution problems and take advantage of its valuable compounds through extraction and production of value-added products [4,5].

Treatment of food processing waste is an important environmental issue; byproducts, such as peels and seeds, can be used as good sources of phenolic and bioactive compounds instead of being disposed of [6]. A significant amount of research has been conducted on extracting and isolating bioactive compounds from plants and plant by-products [6,7,8,9,10,11,12,13]. These compounds can be found in various parts of the plant at different concentrations, and selecting the appropriate extraction method is critical to maximizing the yield [14].

Microwave-assisted extraction (MAE) contributes to extracting bioactive compounds from fruits using microwave radiation. It is a rapid and efficient technique that uses microwave energy to heat the solvent and accelerate the extraction process, which can yield high-quality extracts with high levels of bioactive compounds. It has several advantages over traditional extraction methods, including reduced extraction time, lower solvent consumption, and improved extraction efficiency [15]. In the case of peaches, MAE can be used to extract various bioactive compounds, such as polyphenols and carotenoids [16]. It has potential applications in the food, pharmaceutical, and cosmetic industries, where peach-derived bioactive compounds can be used as functional ingredients [4].

In recent years, there has been a noticeable shift in the food industry towards substituting synthetic compounds, such as colors, stabilizers, and preservatives, with plant-derived extracts. This trend has been largely driven by a surge in consumer demand for healthier and more sustainable food options. The incorporation of plant extracts into yogurts has the potential to enhance the quality and functionality of the final product, as these extracts are rich sources of bioactive compounds [17]. There has already been some research about the usage of plants and their extracts in the dairy industry. Alwazeer and co-workers investigated how plant extracts (specifically, thyme, mint, green tea, ucßkun, and grape seeds) impact the acidification and reducing abilities of *L. del-brueckii* subspecies bulgaricus and *S. thermophilus* in milk, indicating that, when trying to fortify milk with plant extracts as to make various fermented dairy products such as yogurt, one should consider how the acidification and reducing activities of yogurt strains might be affected [17]. Al-Sahlany and co-workers added date juice in yoghurt as a prebiotic, which enhanced the physicochemical characteristics of the products and increased the viability of probiotic bacteria [18]. Furthermore, Bulut and co-workers extracted and evaluated the phenolic content and antioxidant activities of different plants, which then fortificated in set-type yoghurt so as to study its rheological, physicochemical, textural, and sensory properties [19,20].

Mixing of yogurt and peach also has a long culinary history, while research has been conducted on the extraction of valuable compounds from peach by-products [16,21,22,23]. Despite the above, few attempts have been made for the isolation of added-value products from peach and their incorporation into novel, yogurt-type gels. This study aims to extract both phenolic compounds and pectin from peach flesh using microwave-assisted extraction (MAE). The goal is to reduce waste by utilizing lower-grade fruits and to take advantage of eco-friendly extraction techniques, such as (MAE), which consumes less time and energy [24]. The received extracts will be incorporated in strained yogurts as stabilizers and texture modifiers, creating phenolic-rich dairy gels.

## 2. Results

### 2.1. Experimental Design Results and Statistical Analysis

A four-factor Box-Behnken experimental design was employed (pH; liquid to solid (l/s) ratio; microwave power (Watt); Time),as well as three responses (Total phenolic content; ^O^Brix; Particle Diameter (d4.3 reported in μm)), in order to optimize the extraction conditions. The experimental inputs and results are presented in Table 1. The total phenolic content ranged from 3.77 to 39.9 mg/100 mL, expressed as gallic acid equivalents, with the highest values obtained at pH 1. The Brix values ranged from 4 to 13.2%, and the mean particle diameter ranged from 2 to 30. It is common to observe a large range of values in experiments involving natural products due to the inherent variability of the raw materials used. In this case, the heterogeneity of the peaches, which can be affected by factors, such as the tree, maturity stage, and cultivation process, can contribute to the wide range of values observed.

**Figure 1:**
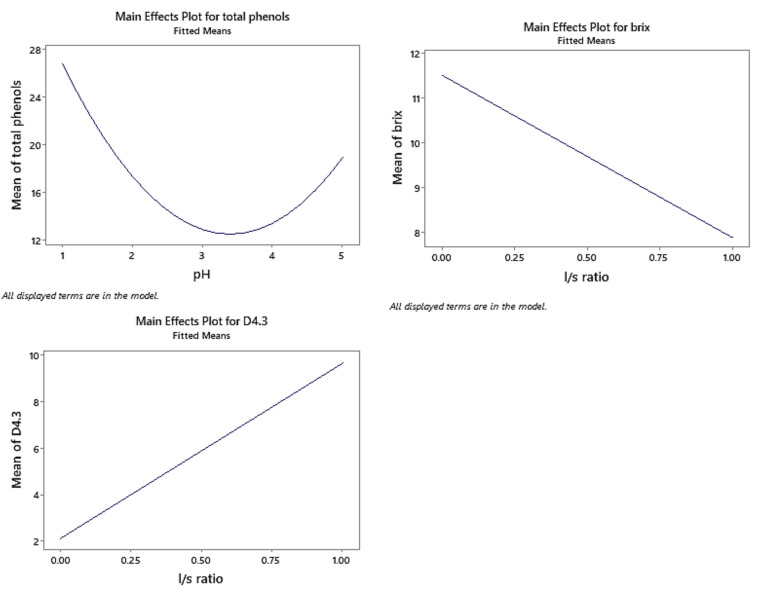
Main effects plot for total phenols, brix, and D4,3.

Figure 1 shows the main effects of the factors studied on each variable. Response surface methodology was used as to investigate the relationship between the variables studied and their responses. The findings demonstrated that the total phenolic content of the peach extracts, measured as gallic acid equivalents, was strongly influenced by the pH level. Interestingly, the highest concentration of phenolic compounds was found at a pH of 1, which suggests that an acidic extraction environment could be more effective in extracting phenolic compounds from peaches. The enhanced extraction at acidic and alkaline conditions is attributed to the acidic (and respectively alkaline) hydrolysis of the bonds that hold the phenolic compounds onto the polysaccharide plant matrix. 

In addition, variations in the liquid-to-solid (l/s) ratio significantly impacted the soluble solid content and particle diameter. Notably, a linear correlation was observed between the l/s ratio and these parameters. The results showed that an increase in the l/s ratio resulted in a decrease in soluble solids content and an increase in particle diameter. These findings highlight the significance of the l/s ratio in optimizing the extraction of bioactive compounds from peaches. Considering the l/s ratio in the extraction process can potentially improve the yield and quality of bioactive compounds from peaches, which may have implications for the development of functional foods and nutraceuticals.

### 2.2. Color and Texture of the Gel Products

**Figure 2:**
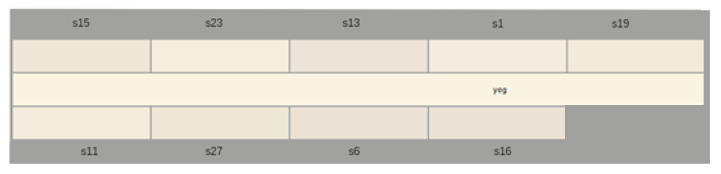
Impact of different extracts on the shade of yogurt in comparison with plain yogurt. The hues are as obtained by the colorimeter.

Table 2 and Figure 2 show the impact of the addition of each extract in plain strained yogurt on the color of the final gel. The colorimetric measurements were conducted using the CIE color scale, with values obtained for L*, a*, and b*, along with color saturation (chroma, c*), hue angle (h°), and whiteness indices obtained from the CIE scale (CIE whiteness) and the whiteness, as per ASTM. The results indicated that all samples were darker and had more red tones than the control while exhibiting less yellow tones. The hue angle decreased, indicating a shift towards more reddish hues. The whiteness indexes were close to the control, but they showed variations due to changes in light scattering caused by the presence of small particles from the extracts in the yogurt. 

**Figure 3:**
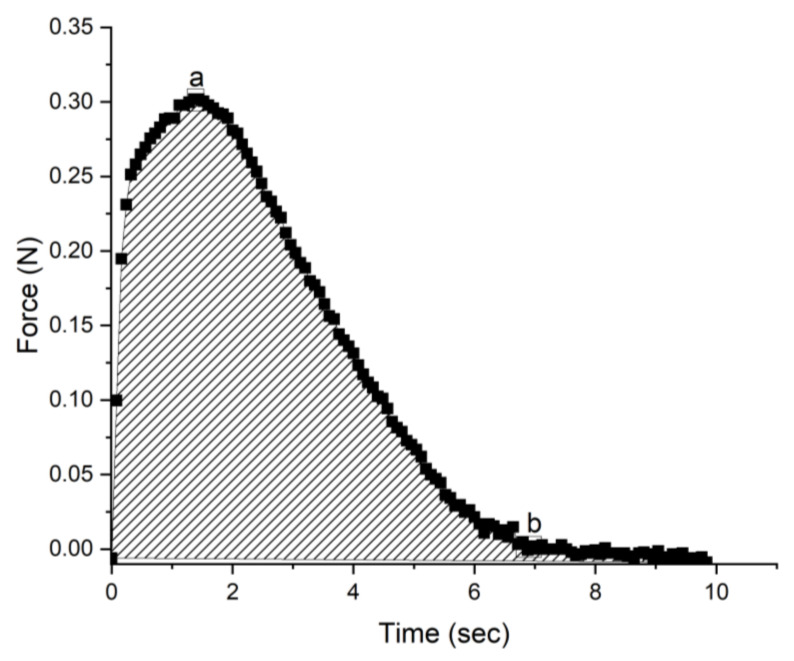
Sample Force–Time plot obtained from the texture analyzer; a is the maximum force, and b represents the filament breakup time. The overall energy expenditure for break-up (work) is the shaded area.

Each measurement was repeated five times, and the parameters for the maximum force, filament break up time, and the deformation area were calculated from the Force–Time figure, as indicated in Figure 3. 

**Figure 4:**
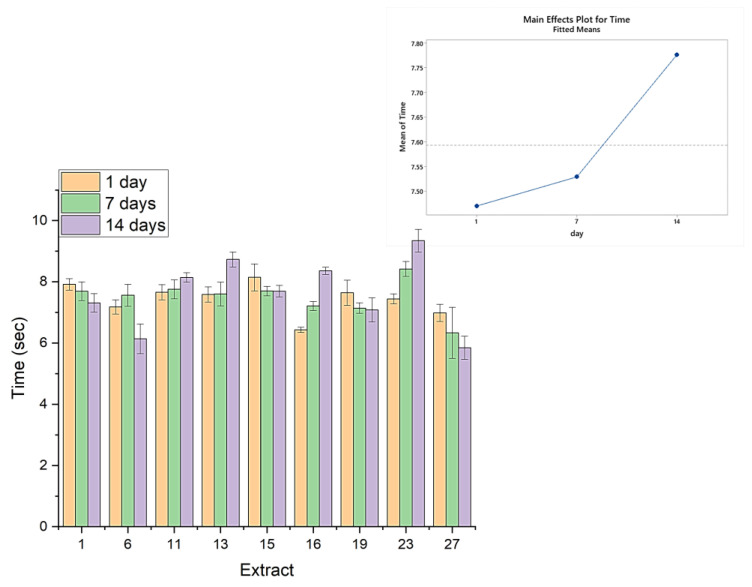
Filament breakup time of yogurts in the first day, as well as after one and two weeks.

Figure 4 shows the filament breakup time for each yogurt measured in the production day after one and after two weeks. Filament breakup time refers to the point at which the yogurt strand being measured by the texture analyzer breaks apart. For samples 11, 13, 16, and 23, filament break up time increases with time, indicating that, in two weeks, the samples were more cohesive. For samples 1,6,15,19, and 27, the cohesiveness slightly reduces over time. An interesting finding is the stability of cohesiveness over time of the products, which is the time required for the filament to break-up, and it remains within about 20% of the initial values, either in an upward or downward direction. Summing this up, the cohesiveness of all samples remains remarkably stable over the gels’ aging of two weeks (break-up times always between 6 s and 9 s), which is close to the expected shelf-life of such products. 

**Figure 5:**
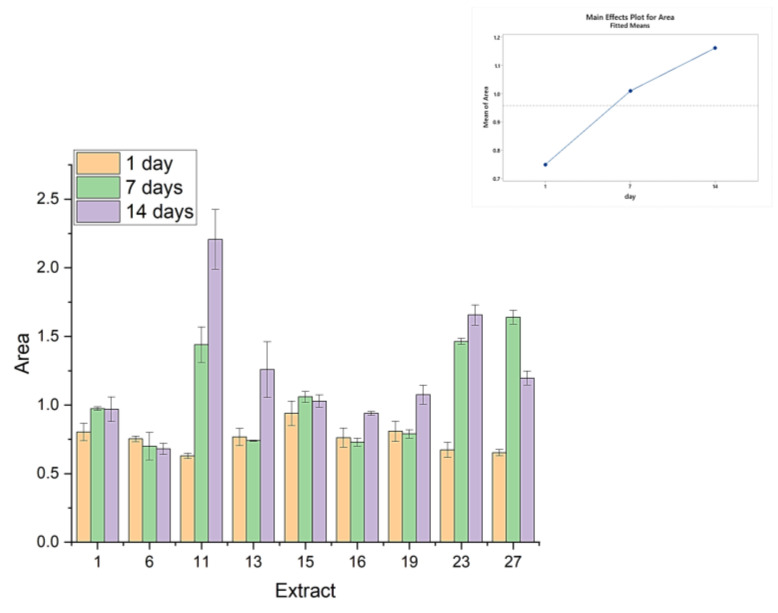
Deformation energy of yogurts in the first day (expressed as area of the Force–Time plot), after one and two weeks.

Figure 5 shows the area of the Force–Time plots of Figure 3. The force needed to deform the products multiplied over the time required for that deformation. As the probe speed is constant, it represents the work required to pull and break up the filament. The samples that have a larger area on a force–time figure are firmer or more resistant to deformation. Conversely, the ones that show a smaller area may be softer or more easily deformable. The work for the deformation of most samples increases with time, indicating that the products become firmer, apparently due to the macromolecular rearrangements in the gel matrix, which increase the material’s reaction to induced deformations. This trend does not apply to samples 6, 15, and 27, which either show their work to break-up reducing over time, either after one or two weeks. These are the samples with the extracts obtained with the highest microwave power (700 W). This energy output is high enough as to partially alter the conformation and self-assembly of the extracted pectins, resulting in the gel losing its firmness over time and requiring less work to break-up.

**Figure 6:**
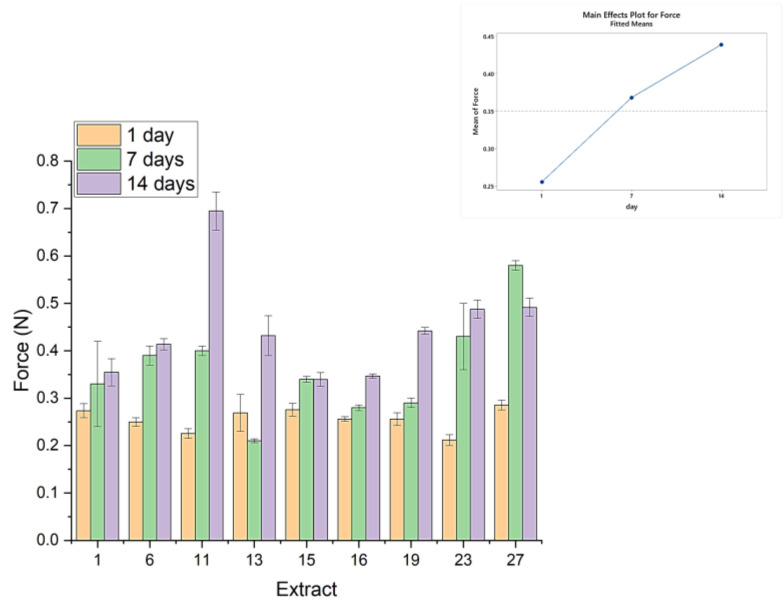
Maximum force obtained before the strand break for the yogurts on the first day, as well as after one and two weeks.

Figure 6 shows the maximum force recorded in the Force–Time plots, and it can be linked to the hardness of each sample. A common trend for all samples is for the hardness of all samples to increase over time, gaining from 20 to 50% of the initial hardness. This is due to the rearrangement of the pectin and the protein macromolecules over time, which enhances the response to mechanical deformations. Samples 11, 15, and 27 (the ones treated at 700 W) again show a rather different behavior: Sample 1 increases in hardness three-fold, that is, from about 0.25 N to 0.7 N; samples 15 and 27 reach a maximal value within one week and then either remain stable (sample 15) or lose hardness (sample 27), and they increase to a maximal value of 0.5 N and then drop to 0.4 N. This, again, highlights the importance of keeping a balance between power output to disrupt the plant matrix without exposing the extracted polymers to very high energies, which could cause them to lose conformation and structure, thus reduce their gel-structuring capacity. 

## 3. Conclusions

Yogurt products have been reinforced with functional ingredients obtained from the microwave-assisted extraction of phenolic compounds and pectin from peach. Surface response analysis revealed that pH and liquid-to-solid ratio significantly affected the extracts. Samples extracted at pH 1 had the highest phenolic content, while increases in the liquid-to-solid ratio resulted in a decrease in soluble solids content and an increase in particle diameter. 

The incorporation of the extracts into yogurts led to a phenol-enriched product of adequate mechanical properties and improved texture compared to the plain strained yogurt. In particular, after two weeks in the refrigerator, the samples were noticeably firmer and more cohesive, unlike strained yogurt, which tended to deteriorate within a few days of opening. 

The cohesiveness of all tested samples remained remarkably stable over the gels’ aging of two weeks, which is close to the expected shelf-life of such products. The required work for the deformation of most samples increased over time, suggesting that the products underwent a significant increase in firmness. This can be attributed to macromolecular rearrangements that occurred within the gel matrix, resulting in a greater resistance to induced deformation. The samples stabilized with the extractions of the highest microwave power (700 W) lost their firmness over time, thus requiring less work to break-up.

## 4. Materials and Methods

### 4.1. Materials

De-ionized water had been used for all the studied formulations and experiments. Strained yogurt was provided from Koukakis Farm SA (Kato Apostoli, Kilkis, Greece). Peaches of appropriate quality were provided from the Agricultural Cooperative “Cosmos” (Makrochori, Imathia, Greece). Citric acid was purchased from Merck KGaA (Darmstadt, Germany). HCl was purchased from Chemlab NV (Zedelgem, Belgium), and NaOH was purchased from PENTA chemicals (Prague, Czech Republic). 

### 4.2. Extraction Process

The extraction process was carried out using a domestic microwave oven device with adjustable power (Watt) and time (s). To adjust the pH of the solvent, citric acid was mixed with water to obtain solutions of pH levels 1, 3, and 5. A total of 50 g of peach flesh was cut into small pieces and mixed with the proper citric acid solution in different liquid/solid ratios of 0, 0.5, and 1 mL/g. The mixture was then placed in a domestic microwave oven (Ok, Imtron GmbH, Ingolstadt, Germany) for 30, 165, or 300 s and at a microwave power of 385, 539, or 700 W. The extraction parameters were defined using a Box–Behnken design, shown in Table 3. The perceived extract was homogenized and centrifuged at 9000 rpm for 20 min so as to separate the residual solids. 

### 4.3. Characterization of Extracts

The total phenolic content in the extracts were measured using the Follin–Ciocalteu method using an Alpha Helios UV-Vis spectrophotometer (Thermo Fisher, Waltham, MA, USA), according to the protocol proposed by Kupina and co-workers [25]. A standard curve of gallic acid (R^2^ = 0.996) was used to calculate the total phenolic content of the extracts, which was expressed as gallic acid equivalents. Each measurement was repeated three times, and the average value was used to determine the total phenolic compounds content.

The soluble solid content of each extract was measured using a handheld refractometer with a range of 32 Brix. 

The particle size of the extracts was determined using a particle size analyzer (Ambivalue, EyeTech, Dussen, The Netherlands) based on the principle of laser obscuration. Each measurement was repeated three times.

### 4.4. Incorporation of Extracts into Yogurts

Selected extracts obtained with different pH, time, power, and liquid to solid ratio (l/s) values (as presented in Table 4) were mixed with strained yogurts in a ratio of 70/30 (yogurt/extract). This ratio was chosen, as it would accommodate the highest possible content of the liquid extract without compromising the gelled character of the final product. The formulations were selected on the basis of their extraction yield. The pH of the samples was measured in order to ensure consistency and quality control. All samples were stored in the refrigerator for a period of two week to assess their stability. No samples with liquid-to-solid ratio 0 were used in the yogurts because the absence of citric acid led to lower yield of extract.

### 4.5. Colorimetric Analysis

Colorimetric data of each sample, including L*, a*, b*, C*, and hue angle, as well as the whiteness index, were measured using a non-contact imaging spectrophotometer (MetaVue vs. 3200, X-rite, Grand Rapids, MI, USA). The measurements were conducted on the production day, and each measurement was repeated twice. The light source was configured as a standard illuminant (D65) at a 10-degree viewing angle. L* represents the brightness of the product, while a* and b* indicate the green-red and yellow-blue colors, respectively. C* indicates the vividness of the color, and h^o^ is the hue angle.

### 4.6. Texture Analysis

The gels consistency over the storage period was evaluated using a texture analyzer (TA.XT. plus, Stable Micro Systems, Ltd., Godalming, Surrey, UK). Each sample was placed on a flat block, and a flat aluminium 50 mm probe was positioned on the surface of the sample. The probe was then moved upwards at a rate of 120 mm/min, pulling the adhered gel. The time and force required to break the filament between the probe and the sample were recorded. This process was repeated five times for each sample. The measurements were conducted on the first day, as well as on day seven and day fourteen of the storage period.

## Figures and Tables

**Table 1:** Extracts characterization.

Row	pH	l/s Ratio	Watt	Time (s)	Total Phenols(mg GAE/100 mL)	^O^Brix	*d*_4.3_ (μm)
1	1	1.0	539	165	19.23	7.3	17.44
2	3	0.5	385	300	9.83	12	1.92
3	5	0.5	385	165	22.57	10	2.35
4	3	0.0	700	165	3.77	13	2.97
5	3	0.5	700	300	20.57	11.8	2.69
6	3	0.5	700	30	9.3	8.7	1.89
7	5	0.5	539	30	18.57	7.9	1.94
8	5	0.0	539	165	35.9	13.2	2.79
9	3	0.5	539	165	10.9	8.7	2.68
10	3	0.5	539	165	13.9	8.5	2.88
11	5	1.0	539	165	13.57	10.5	3.62
12	1	0.5	539	300	39.9	13.2	3.12
13	1	0.5	385	165	23.23	10	2.45
14	3	0.5	385	30	13.87	8	21.76
15	3	1.0	700	165	12.7	7.6	30.14
16	3	1.0	539	30	13.57	6	7.08
17	1	0.0	539	165	30.62	15.1	2.10
18	1	0.5	700	165	31.28	11.2	2.32
19	3	1.0	385	165	13.23	6.6	2.10
20	3	0.0	539	30	14.9	11.2	3.48
21	3	0.0	385	165	18.57	11.1	3.34
22	3	0.0	539	300	6.23	4	3.78
23	1	0.5	539	30	16.57	8.8	4.85
24	3	1.0	539	300	14.23	7.9	1.94
25	5	0.5	539	300	14.57	13.2	7.45
26	3	0.5	539	165	17.23	8.2	2.60
27	5	0.5	700	165	8.57	8.2	15.83

**Table 2:** Effects of different extracts on the color parameters and whiteness of yogurt. R stands for “redder”; B stands for “bluer”; D stands for “darker”.

Sample	L*	a*	b*	C*	h°	CIE-Whiteness	ASTM-Whiteness
Control (set yogurt)	97.47	−0.57	9.00	9.02	93.65	53.56	44.24
1	−2.47 D	2.45 R	−1.99 B	−1.76 D	−2.61 R	55.96	50.48
6	−5.68 D	1.67 R	−1.79 B	−1.73 D	−1.74 R	46.65	44.31
11	−2.34 D	0.65 R	−0.66 B	−0.68 D	−0.63 R	50.11	43.95
13	−5.23 D	2.39 R	−2.09 B	−1.87 D	−2.56 R	49.25	46.45
16	−5.54 D	1.39 R	−1.67 B	−1.64 D	−1.42 R	46.39	43.93
18	−5.26 D	1.86 R	−1.55 B	−1.46 D	−1.94 R	46.61	43.79
19	−2.67 D	0.72 R	−0.88 B	−0.89 D	−0.70 R	50.21	44.52
23	−1.63 D	0.74 R	−0.78 B	−0.80 D	−0.72 R	52.59	45.68
27	−4.37 D	0.41	−0.53 B	−0.55 D	−0.39 R	44.04	40.22

**Table 3:** Extraction parameters.

Sample	pH	l/s Ratio	Watt	Time (s)	Sample	pH	l/s Ratio	Watt	Time (s)
1	1	1.0	539	165	15	3	1.0	700	165
2	3	0.5	385	300	16	3	1.0	539	30
3	5	0.5	385	165	17	1	0.0	539	165
4	3	0.0	700	165	18	1	0.5	700	165
5	3	0.5	700	300	19	3	1.0	385	165
6	3	0.5	700	30	20	3	0.0	539	30
7	5	0.5	539	30	21	3	0.0	385	165
8	5	0.0	539	165	22	3	0.0	539	300
9	3	0.5	539	165	23	1	0.5	539	30
10	3	0.5	539	165	24	3	1.0	539	300
11	5	1.0	539	165	25	5	0.5	539	300
12	1	0.5	539	300	26	3	0.5	539	165
13	1	0.5	385	165	27	5	0.5	700	165
14	3	0.5	385	30					

**Table 4:** Extracts incorporated in strained yogurts.

Sample	Extract pH	l/s Ratio	Watt	Time	Yogurt pH
1	1	1.0	539	165	3.92
6	3	0.5	700	30	4.12
11	5	1.0	539	165	4.27
13	1	0.5	385	165	4
15	3	1.0	700	165	4.24
16	3	1.0	539	30	4.27
19	3	1.0	385	165	4.26
23	1	0.5	539	30	4
27	5	0.5	700	165	4.3

## Data Availability

Data will be available upon reasonable request.
